# Use of 3D printed model as a reconstruction aid in the management of an extensive ameloblastoma of the mandible

**DOI:** 10.1002/ccr3.6047

**Published:** 2022-07-11

**Authors:** Walter A. Odhiambo, James M. Gatune, Symon W. Guthua, Chris Muraguri

**Affiliations:** ^1^ Department of Oral & Maxillofacial Surgery, Oral Pathology and Oral Medicine University of Nairobi Nairobi Kenya; ^2^ Kenya Medical Training College Nairobi Kenya; ^3^ Micrive Infinite ‐ Ruaraka Nairobi Kenya

**Keywords:** 3D printing, ameloblastoma, mandible

## Abstract

Ameloblastoma is a benign jaw tumor that can grow to a very huge size with high rate of recurrence. The large tumors pose a challenge during resection and reconstructive surgery. We present a massive ameloblastoma of the mandible that was surgically treated with the aid of 3D printed model.

## Graphical abstract

Using 3D printed model to achieve better results and shorten intra‐operative time in maxillofacial reconstruction.

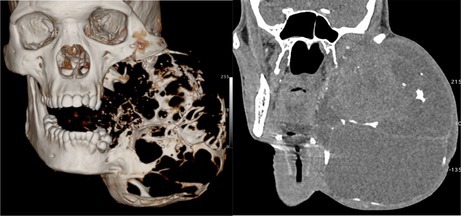

## 1 INTRODUCTION

Ameloblastoma is a locally aggressive benign jaw tumor, with high rate of recurrence. It is an odontogenic tumor arising from residual epithelium of the tooth germ, though etiology remains uncertain.^1^ The tumor has a higher predilection for the mandible (80%–90%) than the maxilla. It is often diagnosed in the 2nd and 3rd decade in the sub‐Saharan African population while a more advanced age is reported in the European literature.

Ameloblastoma is the commonest jaw tumor and is characterized by various histological subtypes. There has been recent reclassification of this tumor into conventional, unicystic, extraosseous/peripheral, and metastasizing (malignant) ameloblastoma. The unicystic variant tends to occur in the younger age groups.^2^, ^3^, ^4^

The management of ameloblastoma is resection of the affected jaw with a safety margin of more than 1.5 cm and reconstruction to restore form and function of the resultant surgical defect. The affected patients among black Africans tend to present to the hospitals late with massive tumors, an observation that has been attributed to a range of factors. Some of these factors include poverty, lack of awareness, traditional beliefs, and possibly asymptomatic nature of the disease.^5^ This late presentation poses a great challenge in the management as it is often characterized by the difficult intubation during general anesthesia that at times require tracheostomy. There is also a threat of damage to major blood vessels and nerves within the proximity as well as reconstruction challenges due to the huge defect created after tumor resection. Extensive tumors may require multiple or staged surgical procedures that at times involve initial reconstruction with titanium implant and later on bridging of the continuity defect with a bone graft. There is usually a lot of time spent intra‐operatively when trying to shape the reconstruction implant into the correct anatomical position. The use of 3D printed models can allow for pre‐operative bending of plates into the desired shape, thus reducing the time spent in surgery.

We present a case of an extensive ameloblastoma in a middle‐aged male that was managed with the aid of 3D printed models.

## 2 CASE DESCRIPTION

**FIGURE 1:**
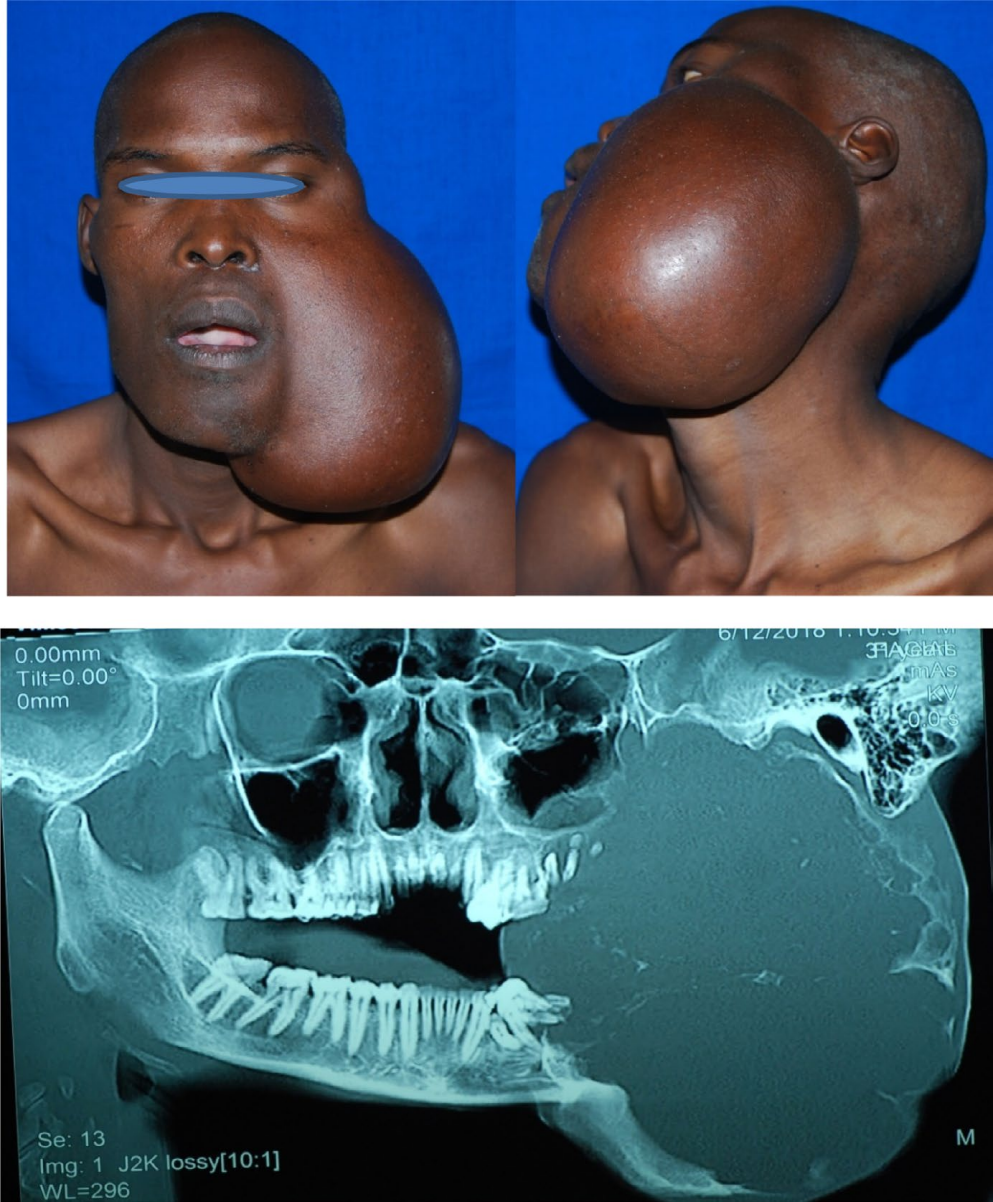
Clinical photographs and CT‐OPG of a patient with ameloblastoma at presentation

A 31‐year‐old male reported to the University of Nairobi Dental Hospital in June, 2018, complaining of massive swelling on the left side of the face. He gave a history of having been well until 2012 when he noticed a swelling after a tooth (38) extraction. The painless swelling had been slowly increasing in size; the associated teeth progressively became mobile and fell off. He had lost about 6kgs over the last 1 year (Figure 1).

Past dental history was significant for tooth extraction one month before he noticed a small swelling at the site for which he was put on antibiotics in a peripheral facility without improvement.

**FIGURE 2:**
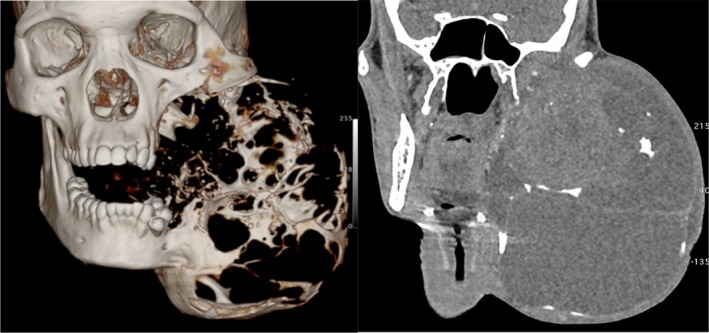
3‐D reconstructed CT scan and coronal view demonstrating the tumor extent

On examination, the patient appeared moderately wasted exhibiting mild conjunctival pallor but no palpable cervical lymph nodes. There was a massive swelling on the left extending from the left temporal region to lower border of mandible measuring19 cm × 16 cm in superficial dimensions. The swelling caused both lingual and buccal expansion with an egg crackling texture in some areas on palpation. A computerized tomographic (CT) scan of the lesion showed an extensive destruction of the mandible and compression of the left maxilla (Figure 2).

**FIGURE 3:**
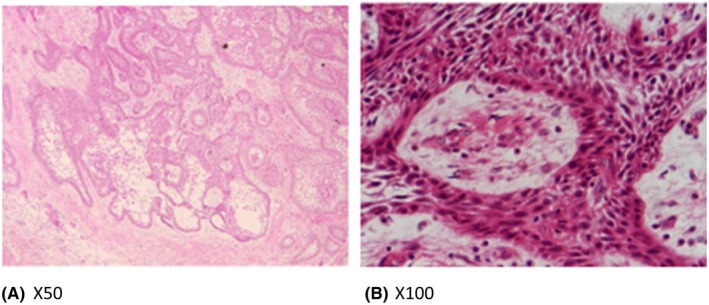
Histopathological picture showing peripheral tall columnar cells with hyperchromatic nuclei surrounding a central mass of stellate reticulum‐like cells features of ameloblastoma

An incisional biopsy was done and showed features of tumor cells suggestive of mixed ameloblastoma (Figure 3).

**FIGURE 4:**
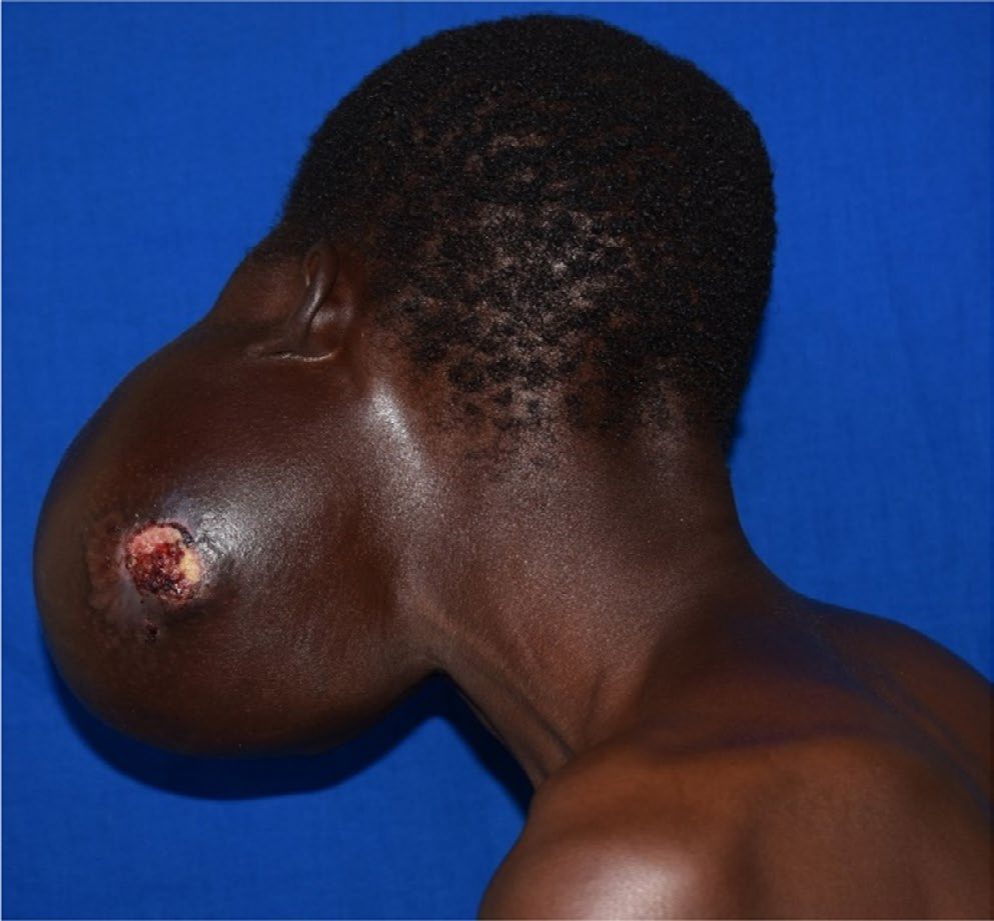
Cutaneous ulceration on the tumor surface 10 months after the initial presentation

The patient had challenges in accessing adequate funds to cater for the cost of surgery and appropriate implant and it took a while for the department to solicit for support for his treatment from corporate partners. He was recalled 10 months later when arrangement for his surgical treatment including sourcing for appropriate reconstruction implant had been finalized. During the waiting period, cutaneous ulceration occurred on the overlying skin thereby creating apprehension of possible malignant transformation. (Figure 4).

**FIGURE 5:**
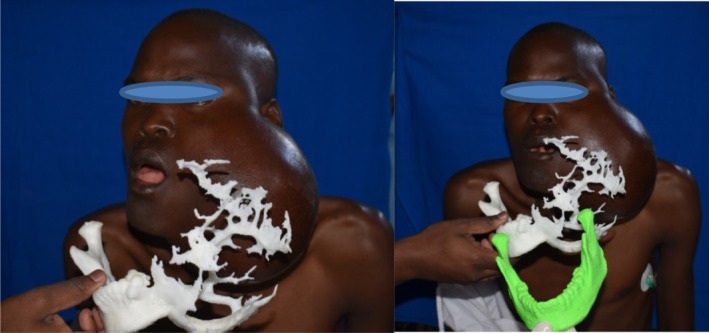
Superimposition of 3D bio‐printed model on the tumor surface(R) and with the reproduced mirror image of the normal side(R)

Preoperative 3‐D bio‐printing was done from the CT scan images and mirror image of the normal side used to generate a stereographic 3D model of a normal mandible (Figure 5).

Routine pre‐operative investigations were done, including full hemogram, urea electrolytes, and creatinine as well as blood grouping and cross matching. The hemoglobin level at admission was 5.7 g/dl, therefore, 3 units of blood were transfused preoperatively and additional 2 units given intra‐operatively.

### 2.1 Surgical approach

**FIGURE 6:**
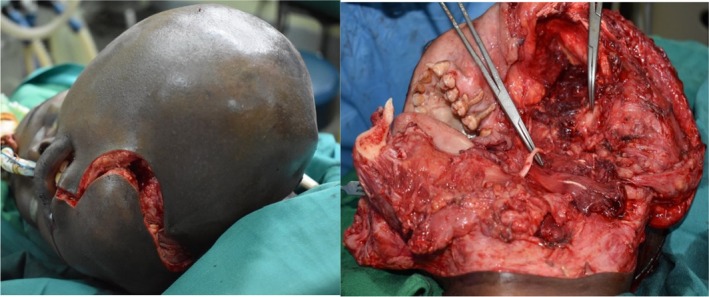
Intraoperative: incision through extended lip split (left), surgical defect after tumor resection with a display of lingual nerve

The patient was prepared in the standard way for craniomaxillofacial surgery and the skin markings made for the planned surgical approach. Through a lip‐split incision that extended to the mental, submandibular, and retromandibular area, the lesion was exposed by a combination of sharp and blunt dissection and tumor successfully resected (Figure 6).

**FIGURE 7:**
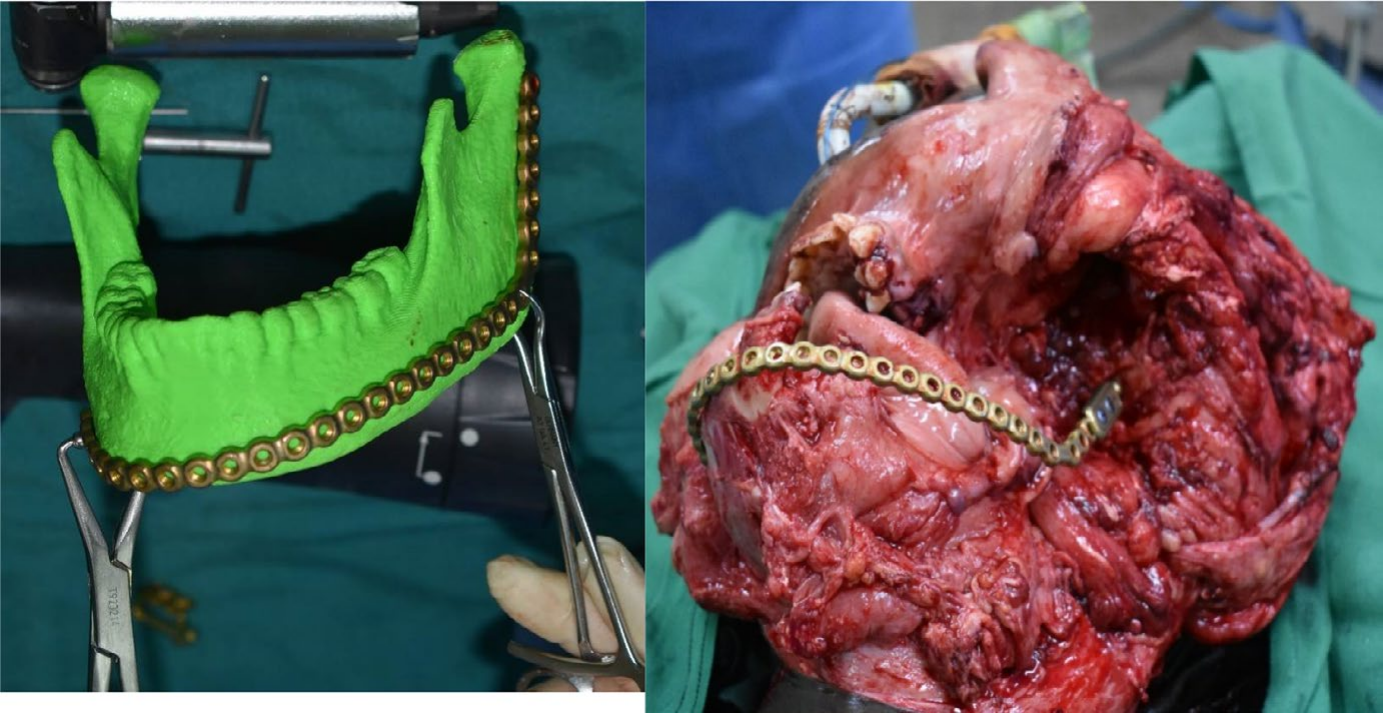
Bending the plate with the aid of the stereographic created 3D model of the mandible (L) and the titanium plate placed in the surgical defect

A titanium mandibular reconstruction plate with condylar head extension was bent into anatomical shape with the aid of 3‐D bio‐printed model of the simulated normal mandible. The plate was then secured to the normal side of the mandible with bicortical screws. The surgical defect was then closed in layers (Figure 7).

**FIGURE 8:**
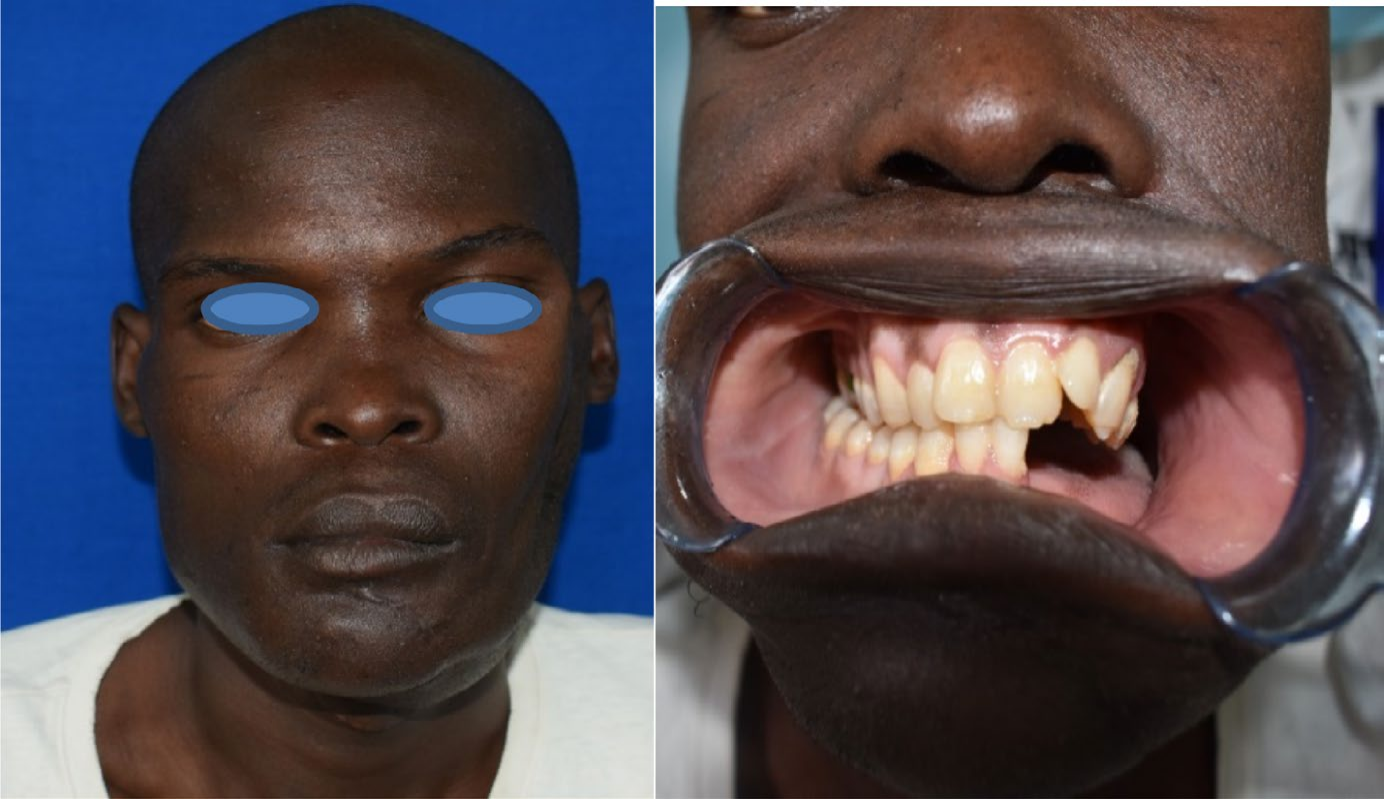
10 weeks post‐operative appearance (left) and satisfactory occlusal relationship of the spared teeth

Post‐operatively, the patient was put on anti‐inflammatory analgesics, antibiotics, and in addition to these routine medication regimes, he was also put on *Ranferon* 30 mg once a day and *Freshubin* 400 mls thrice daily due to the preoperative cachexia. The post‐operative recovery was uneventful and at 3 months follow‐up, the patient was found to weigh 67 kg up from preoperative of 57 kg. The occlusion on the non‐operated side was satisfactory enough for masticatory function, and he awaits a second surgery to bridge the continuity defect with a bone graft (Figure 8).

## 3 DISCUSSION

Ameloblastoma is a locally aggressive benign tumor, with high rate of recurrence (WHO, 2005) but rarely exhibits malignant behavior. This most common odontogenic tumor was previously called adamantinoma and was first identified by Cusack in 1827. It can grow to a very massive size, exhibits high recurrence tendency, and has been reported to recur even more than 5 years after surgery, including recurrence in the bone graft used in the reconstruction.^4^, ^5^, ^6^

It occurs in individuals aged 20–40 years, but a unicystic type occurs more frequently among the adolescent age group. The posterior aspect of mandible is the most common location and the tumor shows no gender predilection, though some authors have reported a higher female incident.^7^, ^8^, ^9^

The management of ameloblastoma remains marginal or en bloc resection with a margin of safety of 1–2 cm, however, some histologic sub‐types have been shown to be less aggressive especially the unicystic variant seen in younger age category. This unicystic type has been treated conservatively in a number of cases often by enucleation with little recurrence.

It has been noted that recurrent ameloblastomas can be diagnosed even 10 years after the first treatment, hence the need for long‐term follow‐up.^8^

The late presentation poses a serious challenge not only in terms of surgical resection of the extensive tumor but also reconstruction of the continuity defect as well as functional rehabilitation. The use of 3D printed helps in highlighting the extent of the tumor as well as appreciating the anticipated challenges that can be discussed with the patient prior to obtaining consent. But what proved useful in this case was the application of the generated 3D stereographic mirror image in creating a near normal mandible that aided in accurate bending of the reconstruction plate to achieve the pre‐pathology normal anatomy.

### 3.1 Conclusion

3D bio‐printing is a useful aid in the treatment planning, surgical reconstruction of extensive jaw tumors and can reduce operating theater time as well as minimize intra‐operative morbidity with improved anatomical profile.

## AUTHOR CONTRIBUTIONS

*Dr. Walter A. Odhiambo* involved in concept and development of the manuscript as well as the management of the patient. *Dr. James Mwangi Gatune* contributed to manuscript writing and management of the patient. *Prof. Symon W Guthua* involved in concept of the 3D and head of surgical team. *Chrirs Muraguri* involved in development of the 3D printing of CT models.

## CONFLICT OF INTEREST

The authors declare no conflict of interest.

## ETHICAL APPROVAL

Ethical clearance was obtained from the UoN Ethics and research committee.

## CONSENT

Patient gave written free un‐coerced consent and was assured of anonymity.

## STATEMENT OF CONSENT

Written informed consent was obtained from the patient to publish this report in accordance with the journal's patient consent policy.

## Data Availability

Data sharing not applicable as no new data generated.
